# Two Tabata cycles in a single training set maximize fat oxidation after exercise in male college students with overweight/obesity

**DOI:** 10.1038/s41598-025-13526-x

**Published:** 2025-09-30

**Authors:** Zhuoyue Cheng, Lixia Liu, Yanbai Han, Hongli Wang, Yongbo Wang, Yiming Han

**Affiliations:** College of Physical Education and Health, Guangxi Normal University, Guilin, Guangxi 541006 China

**Keywords:** Tabata, Energy expenditure, Fat oxidation, Glucose oxidation, Obesity, Weight management, Obesity

## Abstract

Tabata, which is involved 20 seconds of maximum-intensity exercise followed by 10 seconds of complete rest, repeated for 8 cycles totaling 4 minutes, has been identified to enhance energy expenditure and fat oxidation in humans. The study aims to find an optimal Tabata volume for weight loss. 32 male university students with overweight/obesity participated in three tests. Test I consisted of a single Tabata cycle, Test II consisted of two, and Test III consisted of three. Each cycle was separated by a 10-minute interval, and each test was was separated by 7 days. Gas exchange indices were monitored during the last Tabata cycle of the test and the 30-minute recovery period. Subsequently, fat and glucose oxidation amounts, rates, and energy expenditure were calculated. During the 10-minute recovery period, the fat oxidation amounts of Test II (0.80±0.26 g) and Test III (0.87±0.24 g) were higher than Test I (0.50±0.11 g, p<0.001). There was no significant difference between Test II and Test III. During the 20-minute and 30-minute recovery periods, Test II (3.21±0.50 g; 5.04±1.02 g) showed significantly higher fat oxidation amount than Test I (2.47±0.59 g, p<0.001; 4.41±0.98 g, p<0.05) and Test III (2.80±0.43 g, p<0.001; 4.11±0.96 g, p<0.001), there was no significant difference between Test I and Test III (p>0.05). No significant differences in energy expenditure were observed among the three tests during recovery periods (p>0.05). We conclude that two Tabata cycles show the highest fat oxidation amount with the same energy expenditure amount during the recovery period, which is the optimal Tabata volume for weight loss.

## Introduction

In recent years, obesity has risen globally. In 2023, the World Obesity Atlas Federation concluded that more than 50% of the world’s population would be overweight or obese within the next 12 years^1^. China faces a similar obesity crisis. According to the Report on Chinese Residents’ Nutrition and Chronic Disease Status (2020), over 50% of Chinese adults are overweight or obese^2^. Notably, the 20-39 age group demonstrated a relatively rapid increase in obesity rates^3^. In addition, from 2019 to 2021, China’s university students experienced a significant increase in overweight and obesity rates, with male students exhibiting an obesity prevalence of 27.92%, markedly higher than the 13.06% observed in female students‘^4^. This indicates that obesity has emerged as a significant factor effecting the physical and mental health of Chinese male college students.

Prospective cohort studies provide evidence that overweight and obesity are strongly associated with a higher risk of noncommunicable chronic diseases, including cardiovascular disease, type 2 diabetes, cancer, and premature mortality^5^. Research shows that obesity elevates the occurrence of cardiovascular diseases and the risk of mortality among cardiovascular patients^6^. The development of obesity is significantly influenced by genetic and environmental factors, which magnify genetic predisposition to diabetes^7^. Inflammation is a central and reversible mechanism through which obesity increases the risk and progression of cancer, with severe obesity being closely associated with alterations in fat tissue function, adipocyte death, and chronic inflammation, thereby highlighting the health risks that obesity poses to the body^8^. It is clear that obesity has many harmful effects. To tackle this issue, an effective exercise protocol is essential.

Weight management fundamentally depends on achieving an energy balance between caloric intake and expenditure, which explains why weight loss occurs when energy expenditure exceeds intake. Fat oxidation serves as a critical component of energy balance, where enhanced fat oxidation can increase total energy expenditure, thereby facilitating weight loss^9^. Enhanced fat oxidation demonstrates significant metabolic benefits beyond weight management. Robust evidence indicates that elevated fat oxidation rates correlate with improved insulin sensitivity, reduced cardiovascular disease risk, and attenuated inflammatory responses^10–12^. Obese individuals typically demonstrate impaired fat oxidation capacity, which pathophysiologically associates with three key abnormalities: diminished mitochondrial function in skeletal muscle, systemic insulin resistance, and metabolic dysregulation in adipose tissue^13,14^. Therefore, enhancing fat oxidation capacity is critically important for weight management in obese individuals. As a result, traditional weight loss exercise interventions, such as moderate intensity continuous training (MICT), including running, swimming, and cycling, have gained popularity and acceptance. MICT has been shown to increase energy expenditure and decrease fat accumulation^15^. However, these exercise protocols usually demand extended periods, which may not suit the time constraints faced by people nowadays.

High-intensity interval training (HIIT) is an exercise regimen characterized by alternating periods of high-intensity physical exertion and low-intensity recovery phases^16^, offering greater time efficiency and exercise-induced pleasure compared to MICT, which can enhance long-term adherence^17,^^18^. A notable HIIT variant, Tabata training, was developed by Japanese researcher Izumi Tabata and follows a strict protocol of 20-second all-out effort intervals interspersed with 10-second rest periods, repeated over 4 minutes^19^. Unlike traditional HIIT, which operates at 90-110% VO_2_max for 20-30 minutes, Tabata training demands extreme intensity (170% VO_2_max) in ultrashort sessions^20^. This design prioritizes rapid improvements in anaerobic capacity, muscular power, and metabolic rate^21^, making it particularly suited for time-constrained individuals like students or office workers, though beginners should approach it cautiously with modified intensities to avoid overexertion^21,^^22^. In contrast, traditional HIIT’s longer duration and moderate intensity better support cardiovascular endurance and sustained metabolic adaptations^23^. While both protocols are time-efficient, Tabata emphasizes maximized short-term results, whereas traditional HIIT balances intensity with endurance-focused outcomes.

Research demonstrates that Tabata training, like other forms of exercise (both aerobic and anaerobic), induces excess post-exercise oxygen consumption (EPOC), which is a physiological response characterized by elevated energy expenditure after exercise cessation. EPOC is a key mechanism of the delayed effects of Tabata training, as it signifies that the body continues to burn additional energy even after the set has ended, thus elevating the basal metabolic rate^24,25^. Furthermore, the enhanced fat oxidation observed following Tabata training is mediated through multiple metabolic pathways: Notably, post-exercise catecholamine concentrations increase significantly and these neurohormones stimulate lipolysis thus facilitating fat mobilization, with particular efficacy in reducing both subcutaneous and intramuscular fat^26^. In addition, Tabata training enhances fat oxidation through processes such as H+ and lactate clearance, as well as glycogen resynthesis^26^. Moreover, Tabata training significantly boosts interleukin-6 (IL-6) levels in muscles. This exercise-induced cytokine, secreted by fat tissue, activates brown fat thermogenesis (via UCP-1 protein) to enhance energy expenditure, while also providing vascular and neurological protection, collectively improving metabolic health^27^.

Current research on Tabata training primarily focuses on the acute effects of a single Tabata cycle (4-minute protocol). Studies investigating multiple consecutive Tabata cycles within a single training session remain limited. This gap is particularly significant for fat oxidation, which may respond differentially to accumulated Tabata volume. We hypothesize that fat oxidation rates increase with additional Tabata cycles, but reach an optimal threshold beyond which benefits plateau. This study systematically incorporates multiple Tabata cycles into one training session to identify the optimal training volume for maximizing both fat oxidation rates and the delay effect. If the right number of Tabata cycles in a single set are found, we can provide a more personalized training protocol for individuals.

## Methods

This study employed an experimental research method, with 32 male university students with overweight/obesity as participants. After completing the preliminary measurements, including weight, height, body fat percentage, fat mass, lean body mass and resting heart rate, participants performed three Tabata training sessions with a one-week interval between each training session. During the last Tabata cycle of each training session and the following 30-minute recovery period, gas metabolism parameters were continuously measured. These data were then used to calculate the fat and glucose oxidation, and energy expenditure during the exercise and recovery periods, as well as the fat and glucose oxidation rates and energy expenditure rates at the 10th minute, 20th minute, and 30th minute after the completion of the exercise for the three different tests(with the basic data for energy expenditure during the exercise period in Test II coming from the second Tabata cycle and in Test III from the third one).

### Participants

G*Power 3.1.9.7, with an effect size of 0.25, an alpha error probability of 0.05, and a power of 0.8, was used to estimate the sample size. Based on the calculation, the minimum sample size was estimated to be 86, indicating that each test consisting of 29 participants at least. This study utilized a convenience sampling method to recruit participants meeting the following criteria: male university students aged 18-24 years with $BMI\ge 24$ (classified as overweight/obese according to Chinese standards^28^), no regular exercise habits in the past three months, and no history of smoking. Exclusion criteria included metabolic disorders (such as diabetes or dyslipidemia), hypertension, cardiovascular diseases, joint conditions, and students from sports academies. The final sample consisted of 32 overweight/obese male university students, which involved 8 overweight and 24 obese (means ± SD, age 20±1.25 years old; weight 96.06±13.06 kg; height 174.64±7.91 cm; BMI 31.50±4.07 $\text {kg/m}^{2}$; body fat percentage 28.81±4.29%; fat mass 28.38±6.82 kg; lean body mass 67.68±7.73 kg). Participants were asked to avoid intense exercise, smoking, alcohol, caffeine containing drinks, or medication 48 hours before the test. Their diet for the week before the test was recorded, and they were asked to avoid high-sugar and high-fat diets. During the test, participants were required to maintain the same diet as the previous week to eliminate dietary influences. It was also suggested that they keep regular schedules and ensure enough sleep to minimize the potential effect of circadian rhythms and mental state. This study was approved by the ethical review of the Human Ethics Committee of Guangxi Normal University (20231126001). The studies were conducted in accordance with the local legislation and institutional requirements. Informed consent was obtained from all the participants.

### Reproducibility testing

This study did not conduct Cosmed K5 reliability testing. However, the device’s measurement validity has been extensively documented in prior studies^29–31^. Although formal reproducibility assessments were not performed, our team implemented rigorous quality control protocols:

- Strict adherence to manufacturer calibration guidelines;
- Periodic validation using standardized gas mixtures;
- All measurements conducted by a single certified operator;
- Maintenance of consistent environmental conditions.

### Experimental control

The tests were carried out in an indoor environment with a temperature of 24-$26^{\circ }$C and a relative humidity of 40−60%. Each participant performed the test at least 2 hours after lunch, and all tests started at 2:00 p.m. A uniform lunch was provided for each participant, containing rice, beef, vegetables, and a cup of yogurt.

### Experimental procedures

**Fig. 1:**
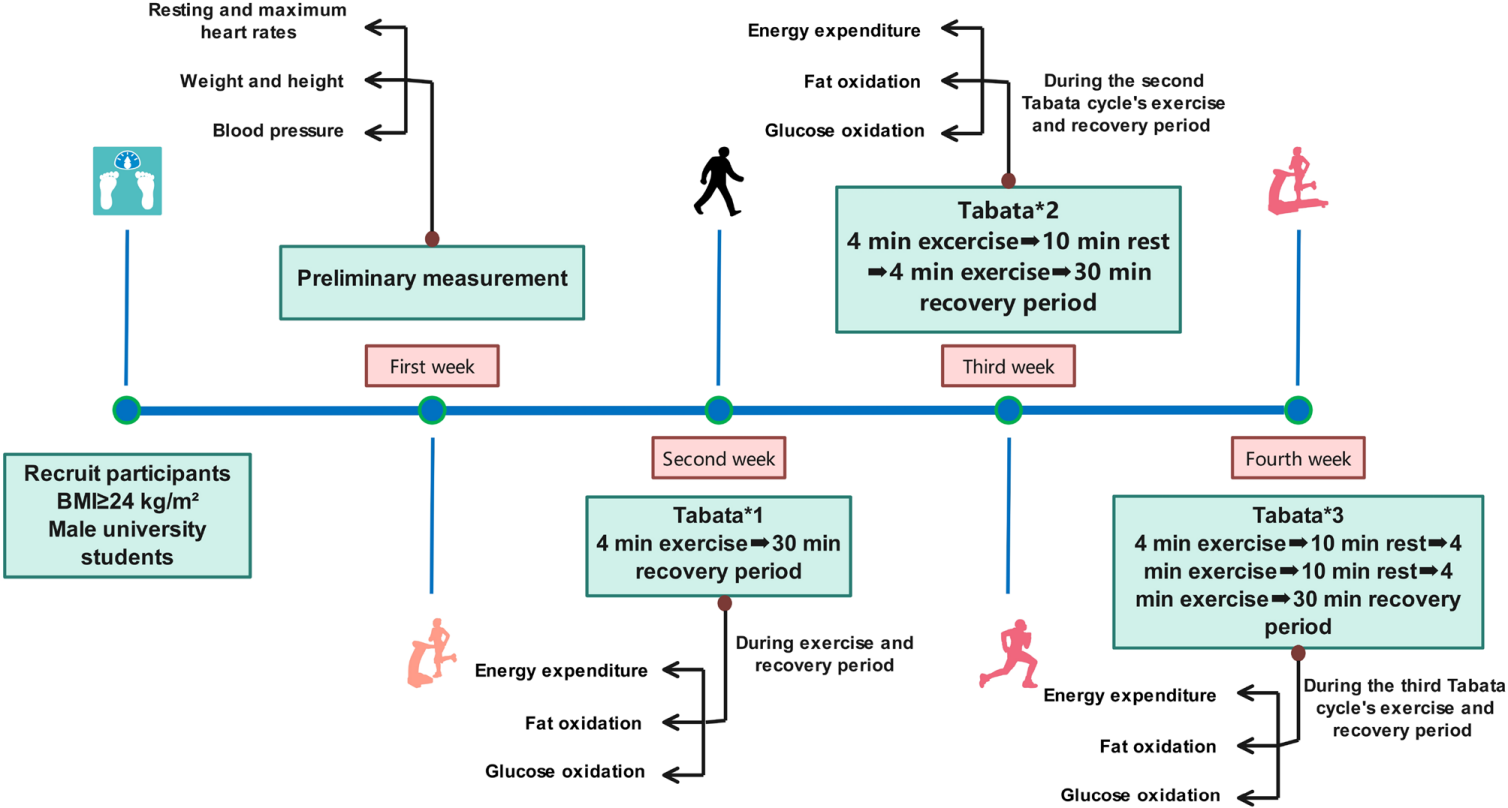
Experimental procedure chart(n=32).

All 32 participants performed three Tabata training sessions of Test I, Test II, and Test III, with one-week between each session. At the start of the experiment, each participant engaged in 10-minutes of sedentary rest. During this period, the staff thoroughly explained and demonstrated to participants: 1) the specific exercises and training procedures included in the Tabata protocol; 2) guidelines for properly wearing the Cosmed K5 device; 3) participants were fitted with heart rate monitors to track exercise intensity. Before the formal training, a 1-minute warm-up which included dynamic stretching of the lower limbs and mobilization of the knees and ankles were done. Participants were then equipped with a cardiopulmonary testing device (Cosmed K5, Italy), which monitored gas exchange indices during the last Tabata cycle of the test and the 30-minute recovery period. A Tabata cycle consists of 4 sets of high leg raises and 4 sets of open and close jumps, totaling 8 sets. Each set lasted 20 seconds with a 10-second rest interval between sets, for a total training duration of 4 minutes. During Test I, participants completed one Tabata cycle. During Test II, they performed two cycles with a 10-minute rest period between each. Test III involved three cycles, also with a 10-minute rest interval between each cycle. (Fig. 1)

### Preliminary measurement

Preliminary measurements included weight, height, body fat percentage, fat mass, lean body mass and resting heart rate. BMI was calculated by dividing body weight by the square of the height ($\text {kg/m}^{2}$). Body fat percentage, fat mass, lean body mass were measured using a body composition analyzer (InBody 770, Korea). Measurements of resting heart rate were taken 2 hours after meals. Maximum heart rates were calculated using the formula (206 - 0.7*age)^32^. Resting heart rate served as the baseline control measurement prior to the experiment, ensuring all participants began testing under standardized resting conditions. Maximum heart rates were used to evaluate fatigue recovery and exercise intensity. To balance Tabata’s intensity requirements with experimental safety, participants were required to reach 85% of their maximum heart rate during training.

### Measure

#### Physiological indices measurement

During the exercise and recovery period, participants wore the Cosmed K5 to continuously measure VO_2_ (L/min) and VCO_2_ (L/min). The calculation formulas are as follows^33,34^:

- Glucose oxidation rate (g/min) = 4.585 * VCO_2_ (L/min) - 3.2256 * VO_2_ (L/min);
- Glucose oxidation amount (g) = Glucose oxidation rate (g/min) * Time (min);
- Fat oxidation rate (g/min)= 1.695 * VO_2_ (L/min) - 1.701 * VCO_2_(L/min);
- Energy expenditure rate (kcal/min)= 3.716 * VO_2_ (L/min) + 1.332 * VCO_2_(L/min);
- Fat oxidation amount (g)= Fat oxidation rate (g/min) * Time (min);
- Energy expenditure amount (kcal)= Energy expenditure rate (kcal/min) * Time (min).

HR was recorded every 1 minute throughout the test period. Rating of perceived exertion (RPE) was assessed using the Borg scale during exercise and at three key time points: at 10, 20, and 30 minutes after exercise to evaluate fatigue recovery^35^.

#### Statistical analysis

All data obtained from the experiment were analyzed using SPSS 26.0 software (Chicago, IL, USA), and results are presented as mean ± SD. The Shapiro-Wilk Test showed that the data were normally distributed. The Mauchly’s test of sphericity showed that the data satisfies the assumption of sphericity. A one-way repeated measures analysis of variance (ANOVA) was used to analyze all the data. If the ANOVA results showed significant differences, Tukey’s HSD Test was used to compare pairwise differences. Effect size was calculated as partial $\eta ^{2}$ ($\eta ^{2}$p), and $\eta ^{2}$p equal to 0.01,0.06, 0.14 presented a small, medium, and large effect respectively^36^. A P<0.05 was considered statistically significant.

## Results

**Table 1:** Substrate metabolism and energy expenditure during exercise and recovery period FO, Fat Oxidation; GO, Glucose Oxidation; EE, Energy Expenditure Data are expressed as mean ± SD. n=32.

Exercise period	Test I	Test II	Test III
FO(g)	1.93 ± 0.41	2.64 ± 0.54	2.94 ± 0.60
GO(g)	11.67 ± 3.05	9.48 ± 2.30	7.79 ± 2.37
EE(kcal)	46.42 ± 6.72	46.98 ± 6.58	46.62 ± 6.35
10-minute recovery period			
FO(g)	0.50 ± 0.11	0.80 ± 0.26	0.87 ± 0.24
GO(g)	19.53 ± 4.81	14.47 ± 2.56	13.86 ± 2.45
EE(kcal)	42.13 ± 5.93	42.29 ± 5.03	42.01 ± 5.31
20-minute recovery period			
FO(g)	2.47 ± 0.59	3.21 ± 0.50	2.80 ± 0.43
GO(g)	25.33 ± 5.97	19.74 ± 3.64	18.38 ± 3.16
EE(kcal)	66.89 ± 7.95	69.07 ± 7.43	68.17 ± 7.88
30-minute recovery period			
FO(g)	4.41 ± 0.98	5.04 ± 1.02	4.11 ± 0.96
GO(g)	27.59 ± 6.40	23.26 ± 4.77	21.82 ± 4.73
EE(kcal)	88.13 ± 13.24	90.39 ± 13.69	87.89 ± 11.93

### Exercise period (Table 1)

**Fig. 2:**
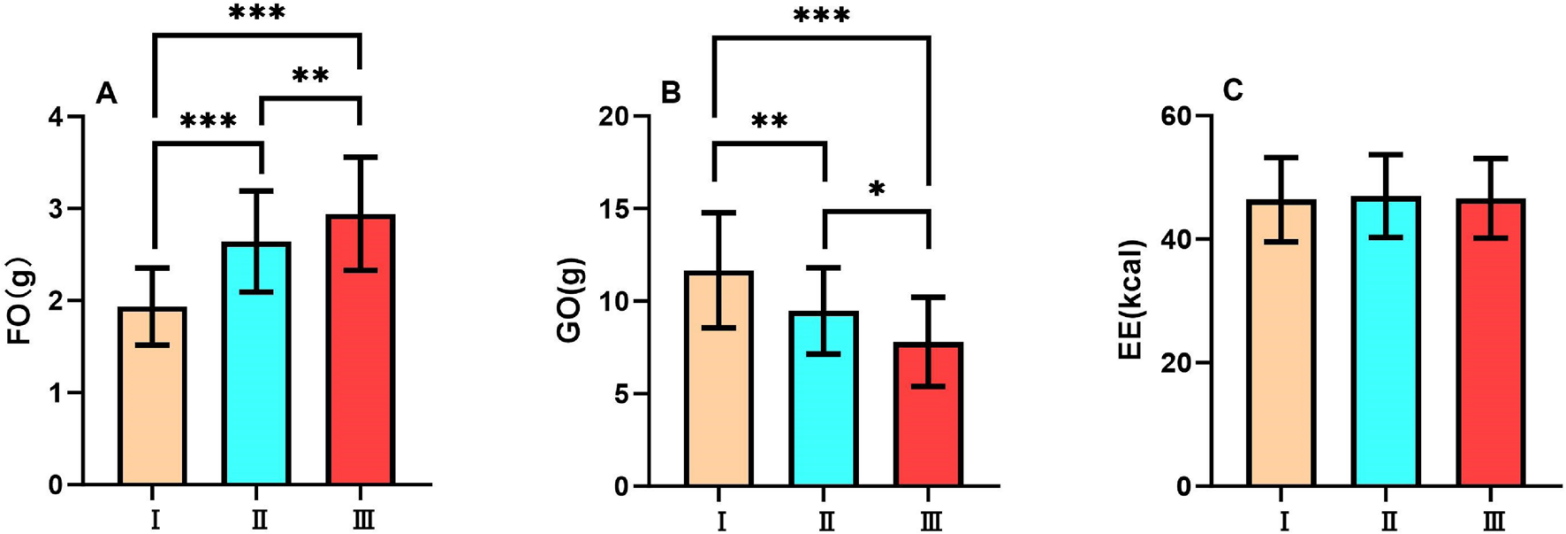
Substrate metabolism and energy expenditure during exercise period FO, Fat Oxidation; GO, Glucose Oxidation; EE, Energy Expenditure of Test I, Test II and Test III during exercise period. Data are expressed as mean ± SD. n=32, *p<0.05, **p<0.01, ***p<0.001.

During the exercise period, there was a significant difference in fat oxidation amount among the three tests (F_2,62_=45.8, p<0.001, $\eta ^{2}$p=0.596). As Tabata cycles performed in a single training set increased, a progressive trend in fat oxidation was observed across the three Tests (all p<0.01). (Fig. 2A)

During the exercise period, there was a significant difference in glucose oxidation amount among the three tests (F_2,62_=37.2, p<0.001, $\eta ^{2}$p=0.545). As Tabata cycles performed in a single training set increased, a progressive decline in glucose oxidation amount was observed across the three tests (all p<0.05). (Fig. 2B)

During the exercise period, there was no significant difference in energy expenditure among the three tests (F_2,62_=0.0427, p>0.05, $\eta ^{2}$p=0.001). (Fig. 2C)

### Recovery period (Table 1)

#### 10-minute recovery period (0-10min following exercise)

**Fig. 3:**
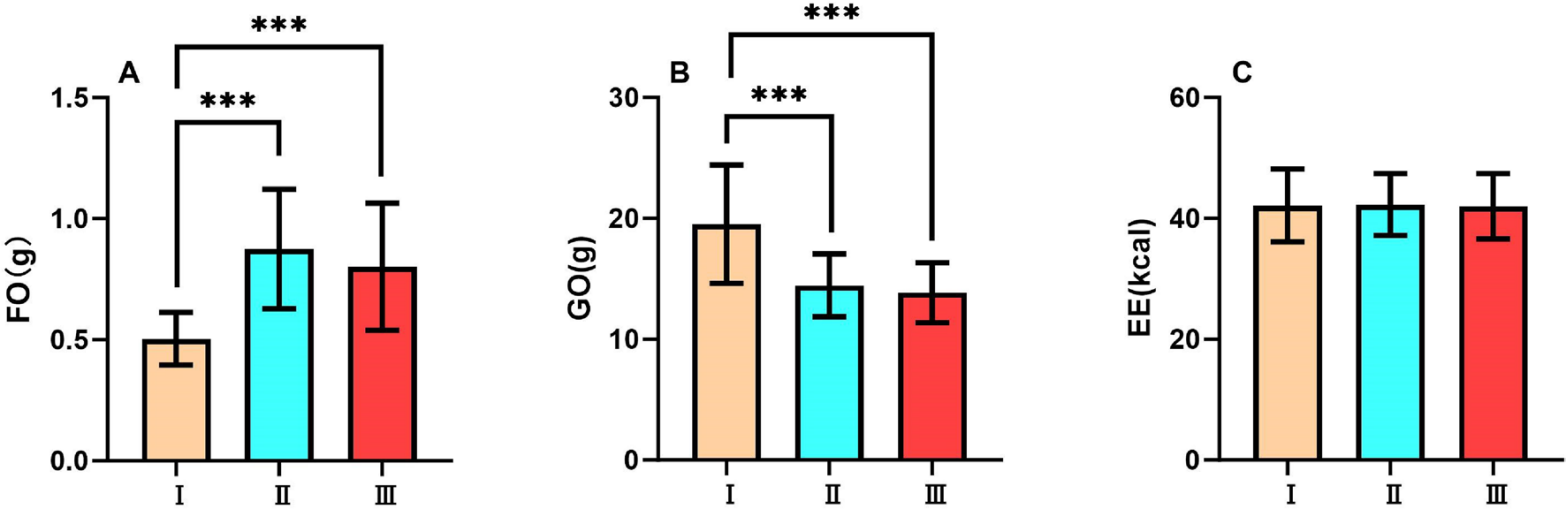
Substrate metabolism and energy expenditure during 10-minute recovery period FO, Fat Oxidation; GO, Glucose Oxidation; EE, Energy Expenditure of Test I, Test II, and Test III in 10 minutes of recovery period. Data are expressed as mean ± SD. n=32, ***p<0.001.

During the 10-minute recovery period, there was a significant difference in fat oxidation amount among the three tests (F_2,62_=28.6, p<0.001, $\eta ^{2}$p=0.480). Test I showed the lowest fat oxidation amount among the three tests (all p<0.001). There was no significant difference between Test II and Test III. (Fig. 3A)

During the 10-minute recovery period, there was a significant difference in glucose oxidation amount among the three tests (F_2,62_=31.9, p<0.001, $\eta ^{2}$p=0.507). Test I showed the highest glucose oxidation amount among the three tests (all p<0.001). There was no significant difference between Test II and Test III. (Fig. 3B)

During the 10-minute recovery period, there was no significant difference in energy expenditure among the three tests (F_2,62_=0.104, p>0.05, $\eta ^{2}$p=0.003). (Fig. 3C)

#### 20-minute recovery period (0-20min following exercise)

**Fig. 4:**
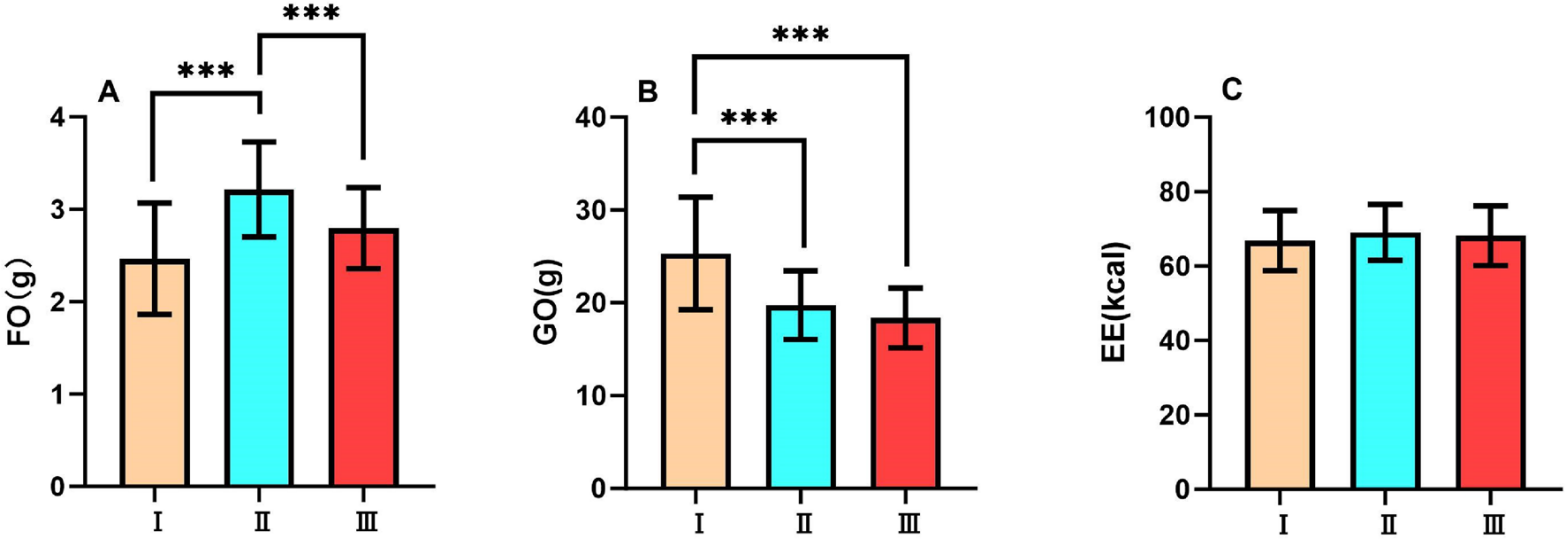
Substrate metabolism and energy expenditure during 20-minute exercise period FO, Fat Oxidation; GO, Glucose Oxidation; EE, Energy Expenditure of Test I, Test II, and Test III during exercise period. Data are expressed as mean ± SD. n=32, *p<0.05, **p<0.01, ***p<0.001.

During the 20-minute recovery period, there was a significant difference in fat oxidation amount among the three tests (F_2,62_=16.4, p<0.001, $\eta ^{2}$p=0.346). Test II showed the highest fat oxidation amount among the three Tests (all p<0.001). There was no significant difference between Test I and Test III. (Fig. 4A)

During the 20-minute recovery period, there was a significant difference in glucose oxidation amount among the three tests (F_2,62_=27.8, p<0.001, $\eta ^{2}$p=0.473). Test I showed the highest glucose oxidation amount among the three tests (all p<0.001). There was no significant difference between Test II and Test III. (Fig. 4B)

During the 20-minute recovery period, there was no significant difference in energy expenditure among the three tests (F_2,62_=0.792, p>0.05, $\eta ^{2}$p=0.025). (Fig. 4C)

#### 30-minute recovery period (0-30min following exercise)

**Fig. 5:**
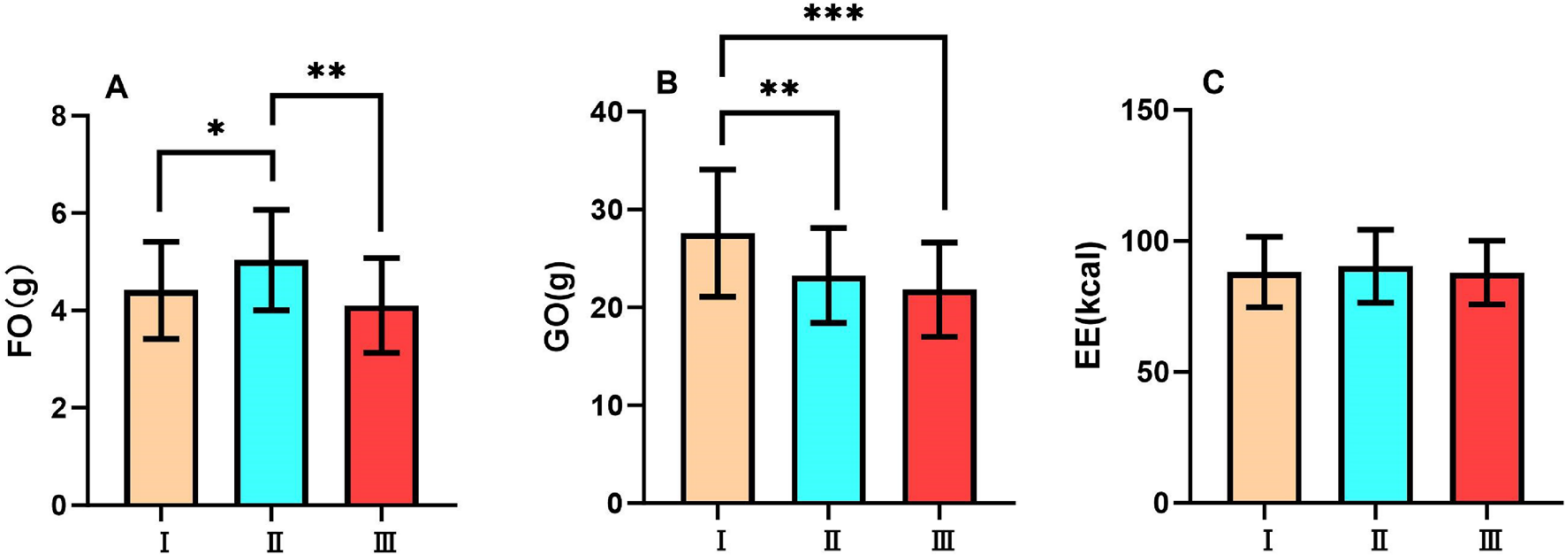
Substrate metabolism and energy expenditure during 30-minute recovery period FO, Fat Oxidation; GO, Glucose Oxidation; EE, Energy Expenditure of Test I, Test II, and Test III in 30 minutes of recovery period. Data are expressed as mean ± SD. n=32, *p<0.05, **p<0.01, ***p<0.001.

During the 30-minute recovery period, the difference in fat oxidation amount among the three tests was statistically significant (F_2,62_=7.89, p<0.01, $\eta ^{2}$p=0.203). Test II showed the highest fat oxidation amount among the three Tests (all p<0.05). There was no significant difference between Test I and Test III. (Fig.5A)

During the 30-minute recovery period, there was a significant difference in glucose oxidation among the three tests (F_2,62_=11.7, p<0.001, $\eta ^{2}$p=0.274). Test I showed the highest glucose oxidation amount among the three tests (all p<0.01). There was no significant difference between Test II and Test III. (Fig. 5B)

During the 30-minute recovery period, there was no significant difference in energy expenditure among the three tests (F_2,62_=0.382, p>0.05, $\eta ^{2}$p=0.012). (Fig. 5C)

### Rates of substrate metabolism and energy expenditure after exercise (Fig. 6, Table 2)

**Table 2:** Rate of substrate metabolism and energy expenditure after exercise at minutes 10, 20, and 30minutes. FOR, Fat Oxidation Rate; GOR, Glucose Oxidation Rate; EER, Energy Expenditure Rate. Data are expressed as mean ± SD. n=32.

	Test I	Test II	Test III
R10th			
FOR (g/min)	0.08 ± 0.02	0.13 ± 0.04	0.13 ± 0.04
GOR (g/min)	0.56 ± 0.12	0.47 ± 0.13	0.40 ± 0.07
EER (kcal/min)	2.60 ± 0.46	2.64 ± 0.34	2.63 ± 0.32
R20th			
FOR (g/min)	0.14 ± 0.04	0.23 ± 0.04	0.17 ± 0.05
GOR (g/min)	0.23 ± 0.05	0.16 ± 0.03	0.20 ± 0.05
EER (kcal/min)	2.22 ± 0.36	2.24 ± 0.28	2.20 ± 0.25
R30th			
FOR (g/min)	0.17 ± 0.04	0.22 ± 0.05	0.17 ± 0.04
GOR (g/min)	0.16 ± 0.07	0.13 ± 0.06	0.15 ± 0.07
EER (kcal/min)	2.02 ± 0.31	2.18 ± 0.30	2.14±0.29

#### At 10th minute after exercise

Fat oxidation rate had a significant difference among the three tests (F_2,62_=34.2, p<0.001, $\eta ^{2}$p=0.525). Test I showed the lowest fat oxidation rate among the three tests (all p<0.05).

Glucose oxidation rate also had a significant difference (F_2,62_=15.8, p<0.001, $\eta ^{2}$p=0.338). Test I showed the highest glucose oxidation rate among the three tests (all p<0.05).

Energy expenditure rate had no significant difference (F_2,62_=0.0880, p>0.05, $\eta ^{2}$p=0.003).

#### At 20th minute after exercise

Fat oxidation rate had a significant difference among the three tests (F_2,62_=28.4, p<0.001, $\eta ^{2}$p=0.478). Test II showed the highest fat oxidation rate among the three tests (all p<0.05)

Glucose oxidation rate had a significant difference (F_2,62_=19.2, p<0.001, $\eta ^{2}$p=0.382). Test II showed the lowest glucose oxidation rate among the three tests (all p<0.05).

Energy expenditure rate had no significant difference (F_2,62_=0.132, p>0.05, $\eta ^{2}$p=0.004).

#### At 30th minute after exercise

Fat oxidation rate had a significant difference (F_2,62_=12.5, p<0.001, $\eta ^{2}$p=0.287). Test II showed the highest fat oxidation rate among the three tests (all p<0.05).

Glucose oxidation rate had no significant difference among the three tests (F_2,62_=1.67, p>0.05, $\eta ^{2}$p=0.051).

Energy expenditure rate had no significant difference (F_2,62_=2.32, p>0.05, $\eta ^{2}$p=0.070).

**Fig. 6:**
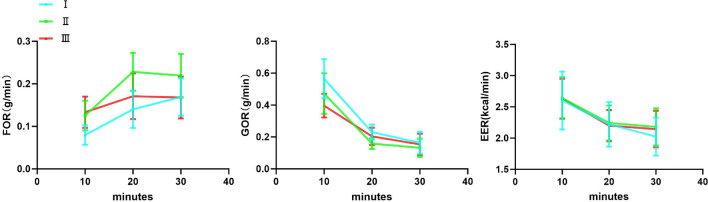
Line chart of substrate metabolism and energy expenditure following Tests I, II, and III at minutes 10, 20, and 30. FOR, Fat Oxidation Rate; GOR, Glucose Oxidation Rate; EER, Energy Expenditure Rate.

### Exercise heart rates and pre-test heart rates (Table 3)

No significant difference was observed among the 85% maximum heart rates of preliminary measurement and the heart rates during the three Tabata cycles (F_3,93_=0.592, p>0.05, $\eta ^{2}$p=0.622). It ensured that all participants have reached the prescribed exercise intensity targets.

No significant difference was observed among the resting heart rates of preliminary measurement and the pre-test heart rates of the three tests (F_3,93_=0.328, p>0.05, $\eta ^{2}$p=0.011). It ensured that all participants initiated testing under standardized resting conditions.

**Table 3:** Exercise heart rates and pre-test heart rates. $HR_T1$: Heart rate during the first Tabata cycle; $HR_T2$: Heart rate during the second Tabata cycle; $HR_T3$: Heart rate during the third Tabata cycle; $HR_P1$: Pre-test heart rate of Test I; $HR_P2$: Pre-test heart of Test II; $HR_P3$: Pre-test heart of Test III. Data are expressed as mean ± SD. n=32.

	HR(bpm)	P value		HR(bpm)	P value
85%HRmax	163.20 ± 0.74	-	Resting HR	86.06 ± 7.11	-
$HR_T1$	163.46 ± 5.91	>0.05	$HR_P1$	85.17 ± 6.97	>0.05
$HR_T2$	164.07 ± 4.93	>0.05	$HR_P2$	85.44 ± 5.77	>0.05
$HR_T3$	164.00 ± 3.57	>0.05	$HR_P3$	86.28 ± 5.63	>0.05

### Rating of perceived exertion (Table 4)

No significant difference was observed among the RPE during exercise (F_2,62_=0.583, p>0.05, $\eta ^{2}$p=0.0182), at the 10th minute after exercise (F_2,62_=0.985, p>0.05, $\eta ^{2}$p=0.031) of the three tests.

The RPE at the 20th minute after exercise had a significant difference among the three tests (F_2,62_=3.45, p<0.05, $\eta ^{2}$p=0.100). There was no significant difference between Test I and Test II (P>0.05). There was a significant difference between Test I and Test III (P<0.05); There was a significant difference between Test II and Test III (P<0.05).

The RPE at the 30th minute after exercise had a significant difference among the three tests (F_2,62_=12.6, p<0.05, $\eta ^{2}$p=0.289). There was a significant difference between Test I and Test II (P<0.05). There was a significant difference between Test I and Test III (P<0.001); There was no significant difference between Test II and Test III (P>0.05).

**Table 4:** Rating of perceived exertion R10th, At 10th minutes after exercise; R20th, At 20th minutes after exercise; R30th, At 30th minutes after exercise Data are expressed as mean ± SD. n=32.

	During exercise	R10th	R20th	R30th
Test I	17.23 ± 0.42	11.27 ± 0.96	10.23 ± 0.73	9.23 ± 0.45
Test II	17.09 ± 0.60	11.36 ± 1.11	10.27 ± 0.81	9.59 ± 0.65
Test III	17.14 ± 0.87	11.59 ± 1.19	10.59 ± 0.72	9.77±0.42
P value	>0.05	>0.05	<0.05	<0.05

### VO_2_ and VCO_2_ during exercise and recovery periods (Fig. 7, Table 5)

**Table 5:** VO_2_, VCO_2_ and respiratory exchange ratio during exercise period and after exercise at minutes 10, 20, and 30minutes. RER, Respiratory exchange ratio Data are expressed as mean ± SD. n=32.

Exercise period	Test I	Test II	Test III	P value
VO_2_ (L/min)	2.57 ± 0.30	2.57 ± 0.29	2.34 ± 0.23	<0.01
VCO_2_ (L/min)	2.73 ± 0.47	2.30 ± 0.32	2.04 ± 0.21	<0.001
RER	1.06 ± 0.10	0.89 ± 0.06	0.87 ± 0.02	<0.001
R10th				
VO_2_ (L/min)	0.57 ± 0.11	0.54 ± 0.12	0.48 ± 0.16	<0.05
VCO_2_ (L/min)	0.52 ± 0.12	0.47 ± 0.11	0.42 ± 0.13	<0.01
RER	0.92 ± 0.10	0.88 ± 0.07	0.89 ± 0.10	>0.05
R20th				
VO_2_ (L/min)	0.53 ± 0.09	0.53 ± 0.11	0.51 ± 0.11	>0.05
VCO_2_ (L/min)	0.41 ± 0.07	0.41 ± 0.09	0.40 ± 0.10	>0.05
RER	0.77 ± 0.05	0.78 ± 0.07	0.80 ± 0.05	>0.05
R30th				
VO_2_ (L/min)	0.52 ± 0.11	0.44 ± 0.07	0.50 ± 0.10	<0.01
VCO_2_ (L/min)	0.39 ± 0.09	0.33 ± 0.05	0.39 ± 0.06	<0.001
RER	0.75 ± 0.06	0.75 ± 0.07	0.79 ± 0.07	<0.01

#### During exercise

VO_2_ during exercise had a significant difference among the three tests (F_2,62_=7.33, p<0.01, $\eta ^{2}$p=0.191). There was no significant difference between Test I and Test II (P>0.05). There was a significant difference between Test I and Test III (P<0.001); There was a significant difference between Test II and Test III (P<0.001).

VCO_2_ during exercise had a significant difference among the three tests (F_2,62_=32.81, p<0.001, $\eta ^{2}$p=0.514). There was a significant difference between Test I and Test II (P<0.001); There was a significant difference between Test I and Test III (P<0.001); There was a significant difference between Test II and Test III (P<0.001).

The RER during exercise had a significant difference among the three tests (F_2,62_=74.57, p<0.001, $\eta ^{2}$p=0.706). There was a significant difference between Test I and Test II (P<0.001); There was a significant difference between Test I and Test III (P<0.001); There was no significant difference between Test II and Test III (P>0.05).

#### At 10th minute after exercise

VO_2_ at the 10th minute after exercise had a significant difference among the three tests (F_2,62_=3.87, p<0.05, $\eta ^{2}$p=0.111). There was no significant difference between Test I and Test II (P>0.05). There was a significant difference between Test I and Test III (P<0.01); There was no significant difference between Test II and Test III (P>0.05).

VCO_2_ at the 10th minute after exercise had a significant difference among the three tests (F_2,62_=5.53, p<0.01, $\eta ^{2}$p=0.151). There was a significant difference between Test I and Test II (P<0.05). There was a significant difference between Test I and Test III (P<0.001); There was no significant difference between Test II and Test III (P>0.05).

The RER at the 10th minute after exercise had no significant difference among the three tests (F_2,62_=1.669, p>0.05, $\eta ^{2}$p=0.051).

#### At 20th minute after exercise

No significant difference was observed among the VO_2 _(F_2,62_=0.395, p>0.05, $\eta ^{2}$p=0.013), VCO_2 _(F_2,62_=0.137, p>0.05, $\eta ^{2}$p=0.004) and RER (F_2,62_=2.26, p>0.05, $\eta ^{2}$p=0.068) of the three tests at the 20th minute after exercise.

#### At 30th minute after exercise

VO_2_ at the 30th minute after exercise had a significant difference among the three tests (F_2,62_=6.17, p<0.01, $\eta ^{2}$p=0.166). There was a significant difference between Test I and Test II (P<0.001). There was no significant difference between Test I and Test III (P>0.05); There was a significant difference between Test II and Test III (P<0.01).

VCO_2_ at the 30th minute after exercise had a significant difference among the three tests (F_2,62_=10.24, p<0.001, $\eta ^{2}$p=0.248). There was a significant difference between Test I and Test II (P<0.001). There was no significant difference between Test I and Test III (P>0.05); There was a significant difference between Test II and Test III (P<0.001).

The RER at the 30th minute after exercise had a significant difference among the three tests (F_2,62_=5.78, p<0.01, $\eta ^{2}$p=0.157). There was no significant difference between Test I and Test II (P>0.05); There was a significant difference between Test I and Test III (P<0.05); There was a significant difference between Test II and Test III (P<0.05).

**Fig. 7:**
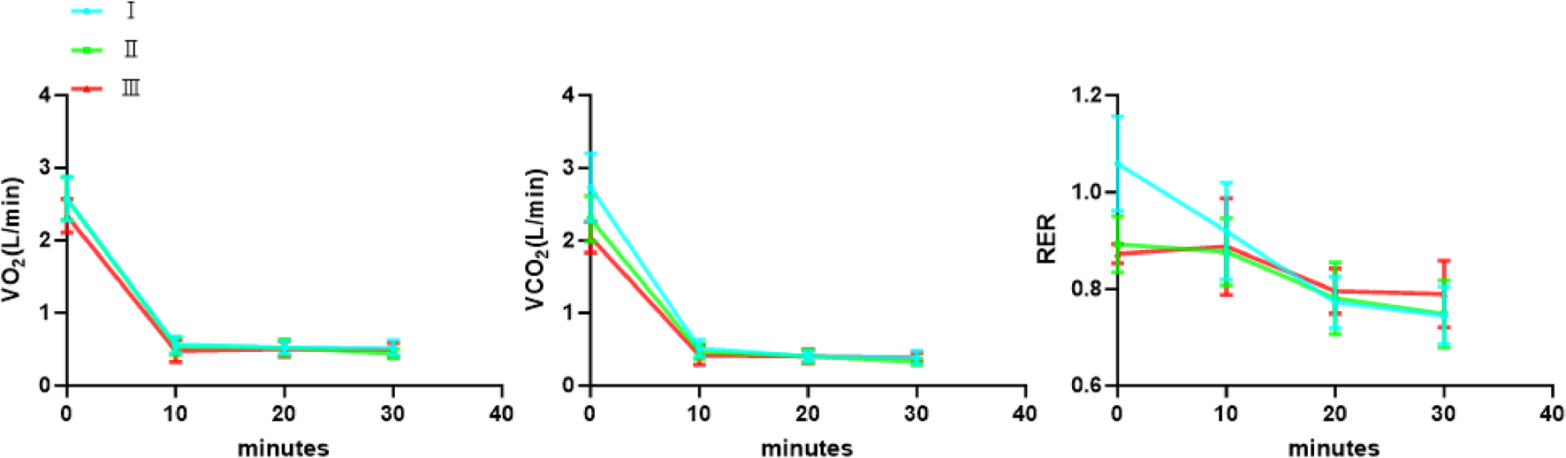
Line chart of VO_2_, VCO_2_ and RER over the exercise and 30-minute recovery period in 3 tests. The data at 0 minute were the average of VO_2_, VCO_2_ and RER during exercise. RER, Respiratory exchange ratio.

## Discussion

### Substrate metabolism analysis during the exercise period

The study shows that when Tabata cycle increases, there is a progressive increase in fat oxidation amount and progressive decrease in glucose oxidation amount across the three tests during the exercise period, suggesting a positive effect of increased Tabata cycles on fat oxidation amount and negative effect on glucose oxidation amount. Regarding substrate metabolism, Tabata training triggers rapid glycogen depletion while simultaneously engaging both anaerobic and aerobic pathways to meet energy demands. During high-intensity intervals, the body initially relies on the phosphagen system (direct ATP-CP utilization) for immediate energy, then progressively recruits glycolytic and fatty acid oxidation pathways^37^. This unique metabolic profile enables Tabata to concurrently enhance fat oxidation and improve insulin sensitivity^38^. Research indicates that Tabata training relies more heavily on carbohydrates as the primary energy source compared to traditional HIIT, particularly during its high-intensity intervals. This metabolic characteristic enables Tabata to rapidly deplete glycogen stores within a condensed time frame^20^. This explains why fat oxidation progressively increases with additional Tabata cycles: as glycogen stores become depleted, the body gradually shifts toward fatty acid oxidation to meet ongoing energy demands^39^. In this process, fat acid is mobilized from adipose tissue into the bloodstream and oxidized by muscle cells, while insulin sensitivity increases and cortisol amount rises^40^, promoting the mobilization and oxidation of fatty acid. Additionally, the activity of enzymes involved in fatty acid oxidation, such as carnitine palmitoyl transferase and acyl-CoA oxidase, significantly increases^41^. These mechanisms indicate that multiple Tabata cycles will promote fat oxidation by increasing training intensity and total volume. The experimental data support this conclusion, demonstrating that multiple consecutive Tabata cycles significantly enhance fat oxidation efficiency compared to a single cycle.

### Analysis of fat oxidation amount during the recovery period

The 10-minute post-exercise recovery period demonstrated progressively increasing fat oxidation rates with additional Tabata cycles, showing a clear dose-response relationship between training volume and lipid metabolism. These findings align with established research demonstrating that during the initial recovery period, HIIT significantly enhances fat oxidation rates compared to moderate-intensity continuous training (MICT). This suggests high-intensity exercise may preferentially stimulate fat oxidation in the post-exercise period^42^. Moreover, As exercise intensity increases (with higher peak power output), the fat oxidation rate correspondingly rises, indicating that high-intensity training can effectively enhance fat oxidation but requires greater training volume to sustain such high-intensity stimulation^43^. This study increased the number of tabata cycles, which increased training volume, so it is reasonable to expect an incremental increase in fat oxidation to occur.

During the 20-, 30-minute recovery period, the training volume corresponding to Test II enabled the body to achieve the best fat oxidation effect and the most significant fat reduction within this period, which meant excessive (Test I) or insufficient (Test III) training volumes were likely to reduce the fat oxidation effect. These results were special. Established research demonstrating a consistent dose-response relationship between training volume and fat oxidation (where greater volume typically yields higher fat oxidation)^44^; during high-intensity exercise, fat oxidation rates remain relatively low, yet post-exercise fat utilization significantly exceeds that of low-intensity training^45^. The distinctive characteristics of Tabata training, specifically its ultra-short duration and maximal-intensity nature, may explain the discrepancy between our findings and prior research. However, another study revealed a distinct pattern: high-intensity training increased fat oxidation rates by 43% during exercise, but elicited a 13% reduction during the recovery phase^46^, which suggested that while high-intensity training enhances fat oxidation capacity, its effects may vary across different physiological phases. This study may explain why excessive Tabata volume leads to reduced fat oxidation during recovery periods.

It is noteworthy that there was no significant difference in fat oxidation between Test I and Test III during the 20-minute and 30-minute recovery periods. This suggests that increasing Tabata volume will not result in higher fat loss efficiency.

In general, these results fully demonstrate that different training intensities and repetitions have a significant impact on post-exercise fat metabolism. An appropriate volume of Tabata training can effectively promote fat oxidation after exercise, while excessive or insufficient training volumes will lead to a reduction in fat oxidation efficiency.

### Analysis of glucose oxidation amount during the recovery period

During the 10-, 20-, and 30-minute recovery period after exercise, Test I had significantly higher glucose oxidation amount compared to Test II and Test III indicating that glucose oxidation amount decreases with the increase in training volume. High intensity training exert significant effects on glucose metabolism. Research demonstrates that HIIT markedly alters post-exercise glucose-regulating hormones and metabolic responses. The active recovery group (ARG) showed a significant decrease in blood glucose levels and increase in insulin levels following maximal exercise, indicating enhanced glucose utilization. Furthermore, this research indicated that intermittent training augments muscular glucose uptake capacity, providing additional evidence that high-intensity training may promote glucose oxidation^47^. This is because after a single high-intensity exercise, the body initially relies on carbohydrates (such as glucose) as the primary energy source. As training volume increases (Test II and Test III), the body increasingly relies on fat and reduces glucose oxidation^48^. The decrease in glucose oxidation amount may reflect a metabolic shift, where the body gradually shifts from primarily re-lying on glucose for energy to relying more on fat^49,50^. This transition typically occurs after high-intensity or prolonged training when the body needs to conserve limited glycogen reserves^51^.

It is noteworthy that there was no significant difference in glucose oxidation between Test II and Test III. These findings align with previous research indicating that after high intensity exercise, the muscle glycogen depletion and reduced glucose oxidation may correlate with increased training volume^52^. Furthermore, while high-intensity training elevates muscle glycogen synthase activity, the glucose oxidation rate may decrease due to preferential utilization of muscle glycogen stores^53^.

In conclusion, these findings collectively suggest that different training intensities and volumes exert a significant influence on energy metabolism in the short term after exercise, particularly in substrate utilization, with clear trends of metabolic shifts from glucose to fat oxidation as training parameters vary.

### Energy expenditure analysis during the exercise period and recovery period

During the exercise period and 30-minute recovery period, despite the increase in Tabata cycles, energy expenditure remained almost unchanged. These results were similar with previous researches. Research indicates that increased training volume typically elevates training load, potentially leading to substantially higher energy expenditure. However, post-exercise recovery energy expenditure may depend more on individual recovery mechanisms and metabolic states. For instance, although HIIT significantly boosts energy expenditure during exercise, post-training recovery energy expenditure can vary considerably among individuals^54^. On the other hand, HIIT reduces the accumulation of muscle metabolites (lactate and phosphocreatine), suggesting enhanced muscular recovery capacity. Nevertheless, this improved recovery may not significantly impact overall energy expenditure during the recovery period^55^. An explanation is that,, although the total exercise load increases with more Tabata cycles, the last Tabata cycle of each test has already maximally mobilized the body’s glycogen reserves and phosphocreatine, leading to stable energy expenditure during the last cycle. This is due to the metabolic demands and energy supply systems are already in a highly efficient and saturated state (a physiological condition where bodily functions achieve optimal equilibrium, representing peak metabolic and biomechanical efficiency)^56^. Adaptation to training intensity may lead to an optimized exercise economy, where the ratio of energy expenditure to exercise performance stabilizes, resulting in almost unchanged energy expenditure for each test^57^.

### Delayed effects of substrate metabolism and energy expenditure after exercise

The study shows the changes in fat oxidation rates for three tests at 10 minutes, 20 minutes, and 30 minutes after exercise. The findings aligned with previous research reporting that fat oxidation rates significantly increase during the 2-hour post-exercise recovery period, reaching a peak within this timeframe. This suggests that while fat oxidation continues to rise during recovery, its rate of acceleration progressively declines^58^. Test I showed a gradual increase in the 30-minute recovery period, which is due to a delayed effect of fatty acid mobilization and oxidation^59^. The fat oxidation rate in Test II and Test III increased from 10 to 20 minutes and decreased again at 30 minutes. This shows that the limited enhancement of fat oxidation rates in the recovery period after increasing Tabata cycles, which may be due to the impact of the participants’ weak cardiorespiratory fitness^60,61^. Test II showed a more pronounced increase in fat oxidation rate throughout the recovery period, especially peaking at 20 minutes, showing that a single training set that includes two Tabata cycles most significantly promotes fat oxidation. Test III, having a relatively stable fat oxidation rate under high-intensity training, did not significantly surpass Test II, which reflects the limited potential for fat oxidation after high-intensity training, possibly related to increased metabolic stress and complexity in energy conversion. In summary, during the recovery period, Test II is most beneficial for fat oxidation, particularly peaking at 20 minutes. The fat oxidation rate of Test I, although increased in the later stages of recovery, is overall lower than Test II and Test III. The fat oxidation rate of Test III was almost the same as that of Test II at 10 minutes, but did not perform as well as Test II thereafter, which may be related to the metabolic load from high-intensity training.

The study also shows the changes in glucose oxidation rates for the three tests at 10 minutes, 20 minutes, and 30 minutes after exercise. All three tests showed gradual decreases in the 30-minute recovery period. The findings aligned with prior research: High-intensity training may increase glycogen synthase activity in muscles, yet glucose oxidation rates could decline due to preferential utilization of muscle glycogen stores. These data suggest that during the early post-exercise phase, participants exhibit robust glucose metabolism, which gradually tapers off as recovery progresses^53^. Our study showed that in the early stages after exercise, participants had enough intense glucose metabolism, which gradually reduced as recovery time extended. At 10 minutes, the glucose oxidation rate decreases with increasing Tabata cycles. At 20 minutes, the glucose oxidation rate was lower in Test II than in both Test I and Test III. At 30 minutes, there was no significant difference in glucose oxidation rate between the three tests. This shows that Test II was weaker in activating glucose oxidation, which is related to changes in exercise intensity^62^. Test I maintained a relatively high glucose oxidation rate throughout the recovery period, especially in the early stages. This shows that after low-volume exercise, the body relies more on rapid glucose metabolism for energy^63^. Test II had the lowest glucose oxidation rate at 20 minutes, indicating a possible earlier shift from glucose to fat metabolism, consistent with its higher fat oxidation rate, reflecting the characteristics of moderate-volume training to trigger metabolic conversion^64^. Test III maintained a relatively stable decreasing trend throughout the recovery period, indicating that high-volume training may maintain a higher metabolic amount over a longer time. In summary, different numbers of Tabata cycles significantly effected glucose oxidation rates during the recovery period. Test I showed higher initial glucose metabolism, which may be related to the body’s dependence on glucose after low-intensity. Test II shifted earlier to fat metabolism, significantly reducing glucose oxidation rates. These trends suggest that post-exercise metabolic pathways were significantly influenced by training volume.

The study showed that the energy expenditure rate of the three tests decreased between 10 and 30 minutes. The number of Tabata cycles had no effect on the energy expenditure rate.

### Research limitations

The study’s exclusive focus on overweight/obese male university students, excluding females, other age groups, and individuals with exercise experience, which limits the generalizability of its findings. While previous studies have validated the reliability of the Cosmed K5^29–31^, and our experimental protocol implemented standardized procedures to ensure data accuracy, the absence of reliability testing in this study may introduce potential measurement variability. Only 1-3 Tabata cycles were examined, leaving the effects of higher training volumes unexplored. The study solely examined acute metabolic responses (30-minute recovery period), failing to assess either the cumulative effects of long-term training on fat oxidation or associated body composition changes.

Future studies should expand participant diversity by including females, various age groups (e.g., adolescents, middle-aged/older adults), and individuals across fitness levels (e.g., trained vs. sedentary populations). Subsequent experimental designs could compare metabolic responses and fatigue accumulation across different Tabata cycle volumes (e.g., 1-3 vs. 4-6 cycles), while long-term intervention trials should track sustained outcomes including body fat percentage, lean mass, and resting metabolic rate.

### Practical implications

Traditional viewpoint held that “greater exercise volume leads to more fat loss,” but this study revealed that excessive volume may paradoxically reduce fat oxidation efficiency. This study proposed an efficient, short-term exercise protocol for overweight/obese individuals: Just 2 Tabata cycles (8 minutes of high-intensity training with 10-minute intervals) could maximize fat oxidation, making it particularly suitable for time-constrained university students or working professionals. For general fitness enthusiasts, this protocol could serve as an effective fat-loss alternative, but required careful intensity management to prevent overexertion or injury.

## Conclusion

This study found that the energy expenditure of the three tests had no significant difference, no matter during exercise or recovery periods. During the exercise period, the more Tabata cycles in a single training set, the more effective the fat oxidation during the exercise period. During the recovery period, two Tabata cycles in a single training set were found to be the most efficient for male university students with overweight/obesity in fat oxidation with the same energy expenditure amount, leading to the best fat oxidation. Regarding the delayed effect post-exercise, a single training set that includes two Tabata cycles showed the best delayed effect among the three tests. Therefore, considering the physical tolerance and time efficiency of male university students with overweight/obesity, a single training set that includes two Tabata cycles is an efficient and suitable exercise method for short-term weight loss for male university students with overweight/obesity.

## Supplementary Information

Supplementary Information.

## Data Availability

Data is provided within the manuscript or supplementary information files.
